# Characteristics of Adenosine-to-Inosine RNA editing-based subtypes and novel risk score for the prognosis and drug sensitivity in stomach adenocarcinoma

**DOI:** 10.3389/fcell.2022.1073688

**Published:** 2022-12-01

**Authors:** Jingjing Pan, Xinyuan Gu, Jing Luo, Xinye Qian, Qiang Gao, Tianjie Li, Longying Ye, Chenlu Li

**Affiliations:** ^1^ The Affiliated Yueqing Hospital of Wenzhou Medical University, Wenzhou, China; ^2^ Department of Laboratory Medicine, The First Affiliated Hospital of Wenzhou Medical University, Wenzhou, China; ^3^ Renji College of Wenzhou Medical University, Wenzhou, China; ^4^ School of Clinical Medicine, Tsinghua University, Beijing, China; ^5^ The First Affiliated Hospital of Wenzhou Medical University, Wenzhou, China; ^6^ Department of Gastroenterology, Affiliated Yueqing Hospital, Wenzhou Medical University, Wenzhou, China

**Keywords:** stomach adenocarcinoma, Adenosine-to-Inosine, survival prognosis, tumor microenvironment, therapeutical sensitivity

## Abstract

Stomach adenocarcinoma (STAD) is always characterized by high mortality and poor prognosis with drug resistance and recrudescence due to individual genetic heterogeneity. Adenosine-to-Inosine RNA editing (ATIRE) has been reported associated with multiple tumors but the potential connection between ATIRE-related signatures and STAD remains unclear. In this study, we comprehensively elevated the genetic characteristics of ATIRE in STAD patients and first screened five vital survival-related ATIRE sites to identify a novel ATIRE-Risk score. Based on the risk scores, we further divided the patients into two different subtypes with diverse clinical characteristics and immune landscapes including immune cell infiltration (ICI), tumor microenvironment (TME), and immune checkpoint expression analysis. The low-risk subgroups, associated with better survival prognosis, were characterized by activated immune-cells, higher immune scores in TME, and down-expression of immunotherapy checkpoints. Moreover, different expressional genes (DEGs) between the above subtypes were further identified and the activation of immune-related pathways were found in low-risk patients. The stratified survival analysis further indicated patients with low-risk and high-tumor mutation burden (TMB) exhibited the best prognosis outcomes, implying the role of TMB and ATIRE-Risk scores was synergistic for the prognosis of STAD. Interestingly, anti-tumor chemotherapeutic drugs all exhibited lower IC50 values in low-risk subgroups, suggesting these patients might obtain a better curative response from the combined chemotherapy of STAD. Finally, combined with classical clinical features and ATIRE-Risk scores, we successfully established a promising nomogram system to accurately predict the 1/3/5-years survival ratio of STAD and this model was also estimated with high diagnostic efficiency and stable C-index with calibration curves. These significant ATIRE sites are promising to be further explored and might serve as a novel therapeutic target for STAD treatment.

## Introduction

Gastric cancer (GC) is the third most common malignant tumor with high morbidity and mortality, of which stomach adenocarcinoma (STAD) is the most common pathological type and generally results in a poor prognosis (Bray et al., 2018). The cause of the disease is still unclear and might be associated with multiple factors, including inherent elements, *H. pylori* infection, and environmental factors (Naumann, 2005). Current primary therapeutic regimen involves surgical resection with adjuvant chemotherapy and radiotherapy, and the development of targeted therapy or immunotherapy also provides new insight to improve the prognosis for STAD (Van Cutsem et al., 2016). However, the prognosis of GC patients with advanced grades remains extremely poor with an average median survival time less than 10–12 months (Song et al., 2017). Despite remarkable improvements have been achieved for the treatment of STAD, especially for targeted or immunotherapy, there are still prominent obstacles such as drug resistance, immune escape, target off and so on (Fuca et al., 2021). Therefore, screening novel indexes with molecular phenotypes is crucial to predict outcomes and curative responses for STAD patients.

Based on transcriptome sequencing, previous publishments have proposed various prognostic models to predict the survival outcomes of GC patients, including immune cells’ characteristics (Li et al., 2021), tumor mutation burden-related scores (Fu et al., 2022), DNA methylation (Zeng et al., 2022) and.etc. Nevertheless, all these models were based on the mRNA expression with the limitations of reliability and reproducibility in clinical practice. This instability results from the regulation of genetic transcription so it is helpful to screen novel types of regulators to overcome this problem. RNA editing is a crucial molecular process through changing specific nucleotide sequences to keep a diversity of gene transcription (Farajollahi and Maas, 2010). Compared to mRNA detection, RNA editing has been reported to possess higher tumor-specificity and independence of inter-individuals at the quantity of RNA and reference gene (Nishikura, 2016).

Interestingly, Adenosine-to-Inosine RNA editing (ATIRE) accounts for about 70% of all RNA editing, referring to a special modification of double-stranded pre-mRNA catalyzed by adenosine deaminases acting on RNA (ADAR) (Nakano et al., 2016). Moreover, recent studies have expounded the characteristics of ATIRE profiling and identified some prognosis-related ATIRE sites in human cancers (Han et al., 2015; Wang et al., 2017). However, the exact relationship of ATIRE with the molecular subtype, chemotherapy response, and prognosis of STAD remains unclear. In this study, we comprehensively elevated the genetic characteristics of ATIRE in STAD patients and divided the patients into two discrete subtypes based on prognosis-related sites. Moreover, we also estimated their clinical characteristics and immune landscapes including immune cell infiltration (ICI), tumor microenvironment (TME), and immune checkpoint expression analysis. Subsequently, the relationship between TMB levels and therapeutic sensitivity to chemotherapy were further estimated, and a novel nomogram scoring system combined ATIRE scores with other classical clinical parameters was successfully constructed to improve the prognostic stratification and facilitate making a clinical treatment decision for STAD patients.

## Materials and methods

### Stomach adenocarcinoma datasets preparation and preprocessing

Transcriptome files with fragments per kilobase million/FPKM values of 374 GC and 33 paraneoplastic tissues were downloaded from The Cancer Genome Atlas (TCGA) datasets (https://portal.gdc.cancer.gov/). We also obtained their corresponding clinical information including age, gender, survival status and time, tumor grades, clinical stages and TNM stages. In addition, ATIRE datasets of these TCGA-STAD patients (285 GC and 33 HC) in stomachic tissues were obtained from the SYNAPSE database with ID: syn2374375 (https://www.synapse.org/#!Synapse:syn4382531). Those patients lack corresponding clinical features and ATIRE sites for analysis were excluded and the ATIRE sites with undefined editing levels in more than 50% of patients were also excluded.

### Identification of variant ATIRE sites in stomach adenocarcinoma patients

To investigate the difference on RNA editing levels between GC and healthy cohorts, we performed the comparative analysis using circulated Wilcox rank test. We also applied the “factoextra” package (https://cloud.r-project.org/package=factoextra/) to conduct the principal component analysis (PCA) to expound the inner heterogenicity for GC patients. The differentially ATIRE sites between GC and HC subgroups were identified with the significance cutoff as absolute fold-change >0.585 (FC > 1.5) and false discovery rate (FDR) < 0.05 with Bonferroni methods. These prominent sites were exhibited in the volcano plots by the “EnhancedVolcano” package (https://github.com/kevinblighe/EnhancedVolcano) and the top50 up- and down-regulated sites were showed in the heatmap using the “heatmap” package (Zhao et al., 2014). The detailed chromosomal locations of significant sites were included in Manhattan chart by the “CMplot” package (Yin et al., 2021).

## Screening prognosis-related ATIRE sites for stomach adenocarcinoma

To accurately screen the prognosis-related ATIRE sites for GC patients, we randomly divided these patients into training and validation datasets with 6:4 ratio. Then, we performed univariate Cox proportional-hazards (Cox-PH) regression analysis for overall survival (OS) via the “survival” package (Hakulinen and Abeywickrama, 1985) in the training datasets. To remove the multicollinearity among parameters, we also performed the Least absolute shrinkage and selection operator (LASSO) regression through the “glmnet” package (Engebretsen and Bohlin, 2019) and signatures with prominent prognostic values were further included into multivariate Cox regression (stepwise model) to obtain the prognosis-related ATIRE sites (Bradburn et al., 2003).

### Constructing ATIRE-based risk score models and associated subtypes

Based on the prognosis-related ATIRE Sites, we further performed the multivariate Cox regression to identify a recapitulatory index (called ATIRE-based Risk Score) to represent the characteristics of these vital sites for STAD. According to the regressive coefficients of multivariate Cox model, the Risk-score was constructed based on the following formula: $Risk\;score=Fre\left(Site1\right)*\beta 1+Fre\left(Site2\right)*\beta 2+\ldots +Fre\left(Site\;n\right)*\beta n$ . In this formula, *Fre*(Site) means the editing frequencies of ATIRE Sites and β means corresponding regression coefficient of each variable. The Risk-score of each GC patient was calculated respectively and the patients were further distributed into high- and low-risk subtypes by setting the median values as the threshold values.

### Evaluating the clinical and immunological characteristics of ATIRE-related subtypes

Subsequently, we systematically evaluated the comprehensive clinical characteristics between the low- and high-risk subgroups including basal demographic features, survival prognosis and pathological stages. To estimate the immunological characteristics of ATIRE-related subtypes in STAD, we further performed the confederate analysis based on multiple immunological aspects, including immune cell infiltration (ICI), tumor microenvironment (TME) and immune checkpoint analysis. For the ICI analysis, we applied the “CIBERSORT” algorithm (Chen et al., 2018) to quantitatively define the infiltration levels of 22 different immune cells with 1000 random permutations. In addition, the ESTIMATE algorithm (Yoshihara et al., 2013) was used to estimate the TME characteristics, including stromal scores, immune scores and tumor purity evaluation. To further evaluate potential curative sensitivity to immunotherapy, we also detected the expression of multiple immune checkpoints between high- and low-risk subtypes including LAG3, HAVCR2, CTLA4, PD1/CD274, and PD-L1/PDCD1.

### Construction and evaluation of ATIRE-Related nomogram scoring system

Combining Risk scores and other clinical phenotypes, including age, gender, pathological grade, clinical stage and TNM stages, we constructed a novel prognostic nomogram scoring system for STAD patients based on the multivariate Cox regression analysis. All these variables were selected with *p* values < 0.05 or clinical experiments and the “rms” package (Zhang and Kattan, 2017) was further applied to construct the nomogram for predicting the one-, three- and 5-years survival ratio for STAD patients. For the evaluation section, the calibration curves with 1-year, 3-years, and 5-years survival were drawn via “calibrate” function of “regplot” package and the Harrell’s C-index was applied to evaluate the stability of this model (Zhang et al., 2018). The “timeROC” package was applied to perform the 1/3/5-years ROC analysis to estimate the predictive value of the nomogram scoring system (Blanche et al., 2013). Finally, clinical application of the nomogram model was assessed by the decision curve analysis (DCA) and the clinical impact curves (CICs) were also plotted by “ggDCA” package (Xiao et al., 2021).

### Identification of DEGs and functional enrichment analysis

The different expressional genes (DEGs) between ATIRE-related groups were identified with absolute fold-change >1 and FDR<0.05. The “ClueGO” plug-in of Cytoscape software (Smoot et al., 2011) and “ClusterProfiler” package were applied to conducted Gene Ontology (GO) and Kyoto Encyclopedia of Genes and Genomes (KEGG) functional enrichment analysis based on above DEGs (Wu et al., 2021). Gene Set Enrichment Analysis (GSEA) was also performed to comprehensively reveal the functional characteristics of ATIRE-related subtypes.

### Relationship of drug susceptibility, tumor mutation burden and ATIRE-based risk score in stomach adenocarcinoma

The mutation data of GC patients was downloaded from the TCGA datasets and corresponding TMB scores were calculated using the “maftool” R package (Mayakonda et al., 2018). The relationship between TMB scores and Risk scores was also evaluated and combined survival analysis was further applied to expound their prognostic value. To further assess the clear therapeutic response of Risk scores to the traditional chemotherapy for STAD, we calculated the half-maximal inhibitory concentration (IC50) of frequently-used chemotherapeutic drugs using the “oncoPredict” package according to the Genomics of Drug Sensitivity in Cancer (GDSC) database (Maeser et al., 2021). In clinical practice, multiple chemotherapeutic schemes had been widely recommended for STAD based on current clinical guidelines (Ikegame and Terashima, 2020), such as Cisplatin, 5-Fluorouracil, Docetaxel, Oxaliplatin, Epirubicin, Paclitaxel and so on. Therefore, the difference of above chemotherapeutic drugs’ IC50 value between low- and high-risk cohorts was identified by Wilcoxon’s test and the results were exhibited in box-line charts using the “ggpubr” package (Whitehead et al., 2019).

### Statistical analysis and ethical statement

This study was approved by the Ethics Committee of the Affiliated Yueqing Hospital of Wenzhou Medical University (ID: 2021063). The statistical analysis was completed in R software (version 4.0.3). The comparison of continuous variables was conducted with Wilcox Rank-Sum test and the survival analysis was performed bu Kaplan-Meier algorithm. The two-tailed adjusted *p*-value <0.05 was considered as statistical significance.

## Results

### Characteristic of ATIRE sites in stomach adenocarcinoma patients

**FIGURE 1:**
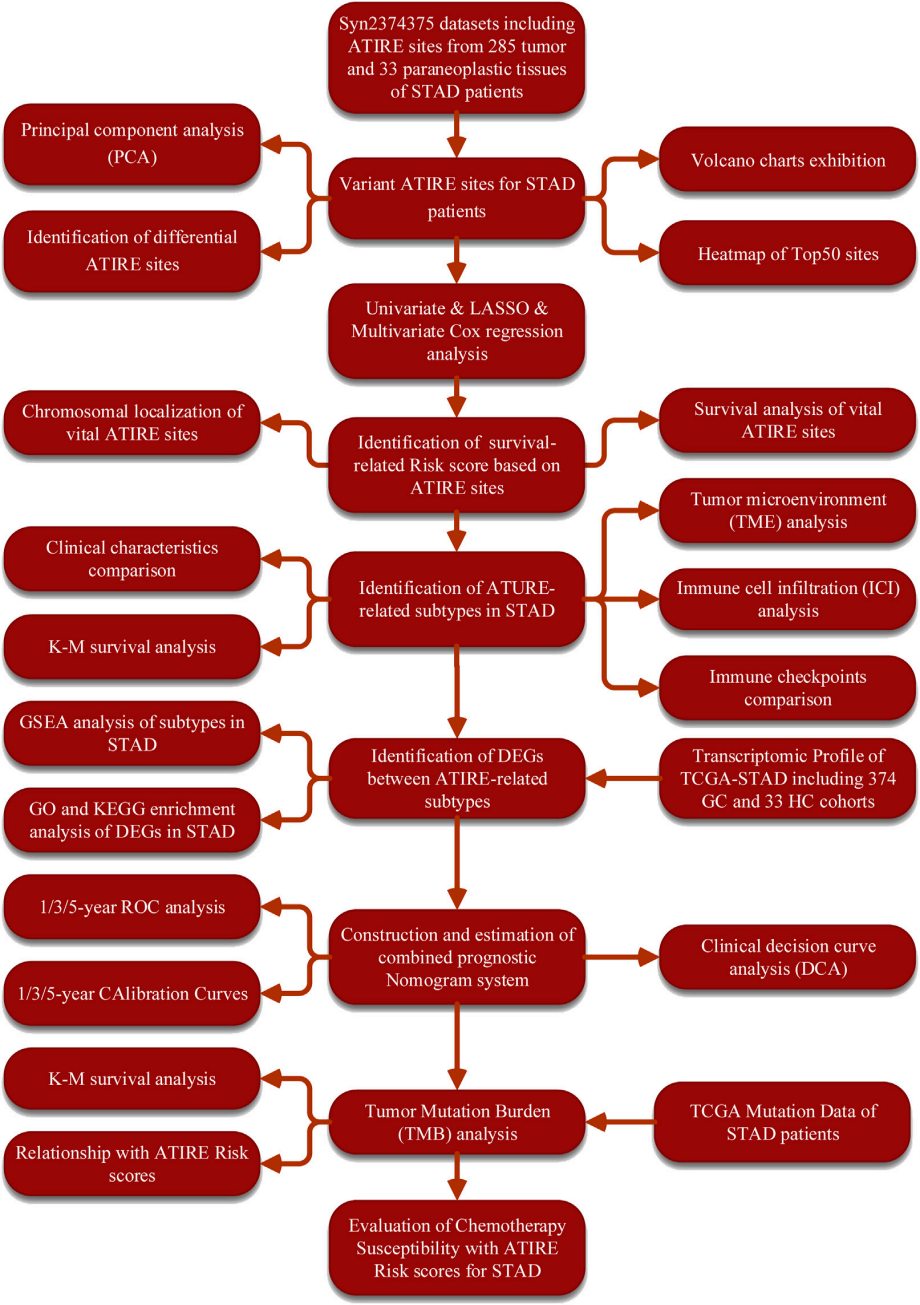
The flow charts of the workflow in our study.

**FIGURE 2:**
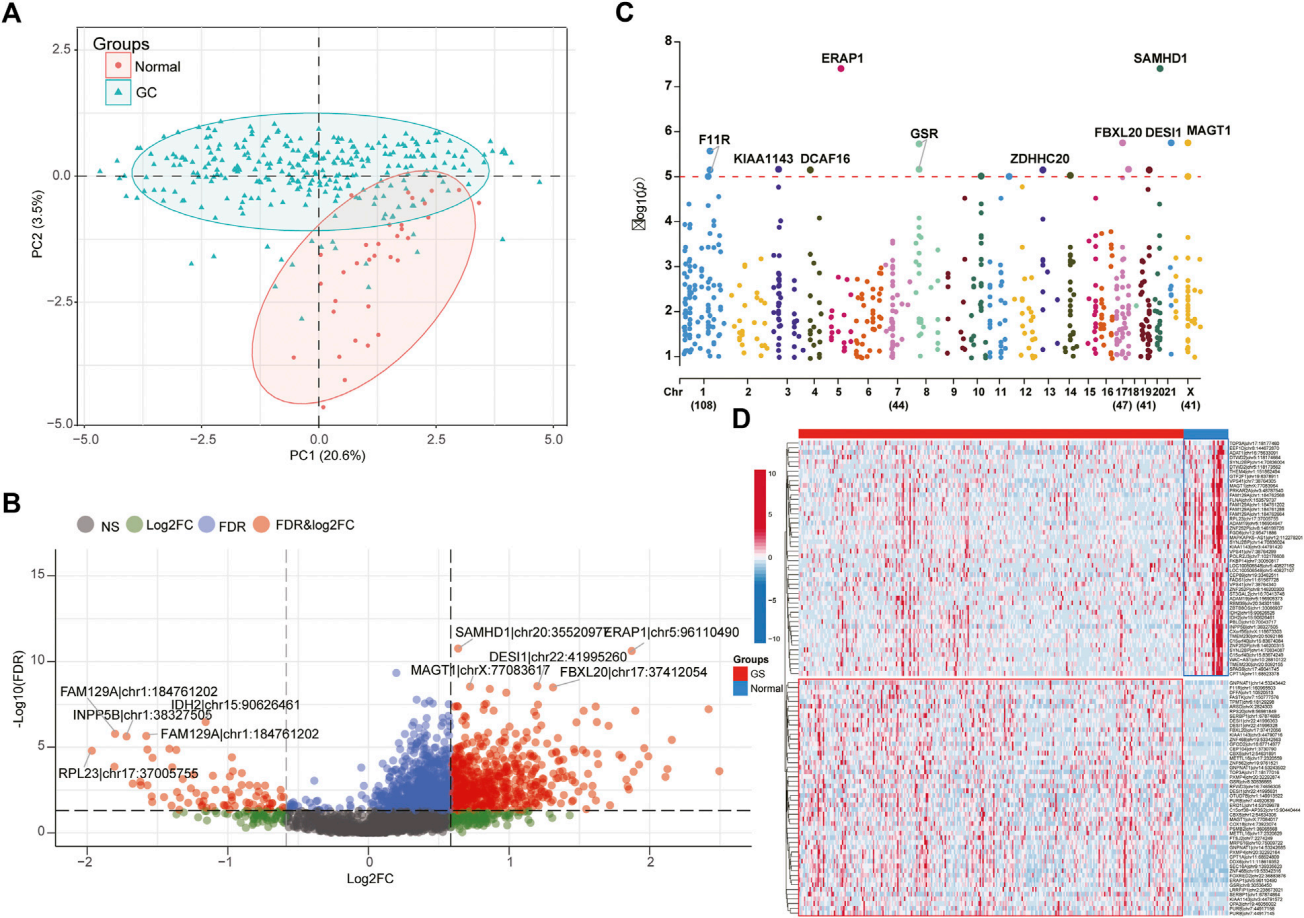
Identification of different ATIRE sites between STAD and HC. **(A)** PCA analysis indicated the HC and STAD patients were significantly divided into two cohorts based on ATIRE sites. **(B)** Volcano plots showing the different ATIRE sites between STAD and HC cohorts. **(C)** The top5 up-regulated and down-regulated sites were displayed in the Manhattan diagram. **(D)** The heatmap revealed the expression levels of top50 up-regulated and down-regulated sites between two groups.

The workflow chart of our study was exhibited in the Figure 1. To elaborate the heterogenicity of ATIRE sites, we performed the PCA analysis and it revealed that GC and HC patients were significantly divided into two distrinct cohorts based on their levels (Figure 2A). In addition, a total of 672 variant ATIRE sites were identified with *p*-value < 0.05 and absolute fold change >1.5, including 594 upregulated and 78 downregulated sites (Figure 2B). The characteristics chromosomal locations of these significant sites were also included in Manhattan chart and we detected that most sites were located in chromosome 1, 7, 17, 19, X with more than 40 sites, of which top10 sites were exhibited with the most significant *p*-value (Figure 2C). The top50 upregulated and downregulated ATIRE sites exhibited obvious difference in two statuses (Figure 2D).

### Subtypes based on survival-related risk score of ATIRE sites and their clinical characteristics in stomach adenocarcinoma

**FIGURE 3:**
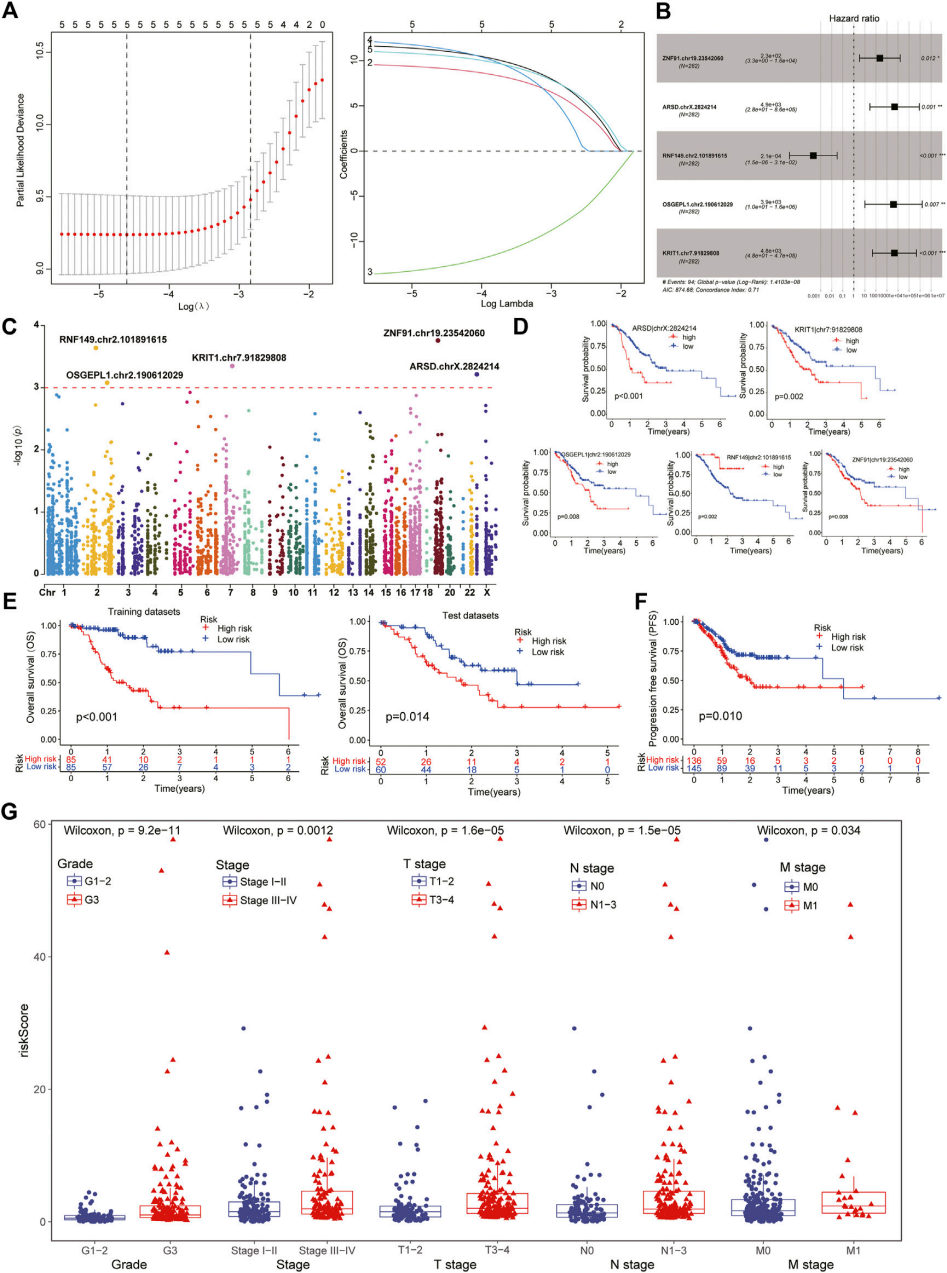
Identification of survival-related ATIRE sites in STAD patients. **(A)** After the univariate Cox analysis, the LASSO regression obtained five hub sites with the lowest Lambda value. **(B)** Five vital survival-related ATIRE sites were identified by multivariate Cox regression analysis, including ZNF91.chr19.23542060, ARSD.chrX.2824214, RNF149.chr2.101891615, OSGEPL1.chr2.190612029 and KRIT1.chr7.91829808. **(C)**. The Manhattan diagram showing the chromosomal locations of above five vital sites. **(D)** Survival analysis indicated that four sites were malgenic sites while one site was protective sites for the prognosis of STAD. **(E)** Subtypes with low-risk scores exhibited better overall survival than high-risk patients in both training and test datasets. **(F)** Progression free survival (PFS) analysis also demonstrated a better prognosis in low-risk patients. **(G)** Clinical comparison showing that higher ATIRE-Risk scores were identified in more severe clinical status including Stage III-IV, Grade III, T3-4, N1-3, and M1.

To further explore and compare the survival characteristics of ATIRE sites in GC patients, we performed a series of analysis to obtain a survival-related Risk score using univariate Cox, LASSO and multivariate Cox regression analysis. It revealed the Risk score was consisted of five sites from the stepwise model with the minimal Akaike Information Criterion (AIC) value, including ZNF91.chr19.23542060, ARSD.chrX.2824214, RNF149.chr2.101891615, OSGEPL1.chr2.190612029 and KRIT1.chr7.91829808 (Figure 3A,B). According to the regressive coefficients of survival-related sites, the Risk score was favourably established using the following formula: $Risk\;score=\mathrm{Exp}\left(ZNF91|chr19:23542060\right)*11.981+\mathrm{Exp}\left(ARSD|chrX:2824214\right)*9.770+\mathrm{Exp}\left(RNF149|chr2:101891615\right)*\left(-14.132\right)+\mathrm{Exp}\left(OSGEPL1|chr2:190612029\right)*\left(12.539\right)+\mathrm{Exp}\left(KRIT1|chr7:91829808\right)*\left(11.310\right)$ . Of this model, four sites were malgenic and one site was protective role, and they were located in chromosome 2, 7, 19, and X respectively (Figure 3C). The K-M survival analysis further demonstrated its predictive capability for GC patients both in malgenic and protective role (Figure 3D).

**FIGURE 4:**
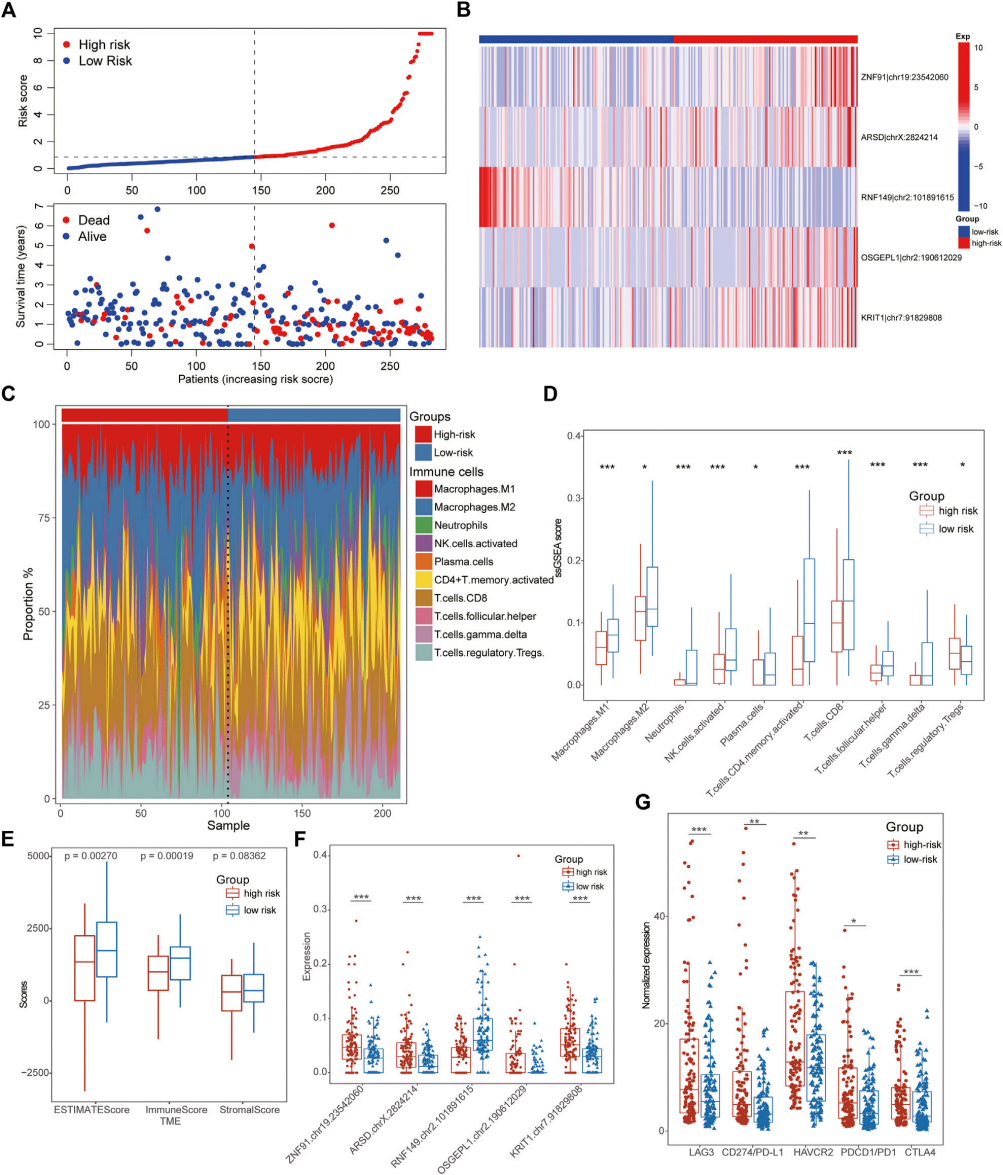
Immunological characteristics of ATIRE-related subtypes in STAD. **(A)** The number of death patients was increased with the increasing of Risk scores. **(B)** The heatmap showing editing frequencies of five hub ATIRE sites between low- and high-risk patients. **(C)** The scale charts showing the different infiltrate ratios of immune cells between low-risk and high-risk subgroups. **(D)** Multiple immune cells were infiltrated in the tissues of low-risk GC patients, such as Macrophages, activated NK cells, CD4^+^ T-cells, CD8^+^ T-cells, Plasma cells and so on. **(E)** TME analysis displayed that the low-risk patients exhibited higher immune and stromal scores than that of high-risk STAD patients. **(F)** Box line diagrams showing the different expression of five hub ATIRE sites between low- and high-risk patients. **(G)** The low-risk patients possessed a lower expression of immune-checkpoints than high-risk groups, including LAG3, HAVCR2, CTLA4, PD1/CD274, and PD-L1/PDCD1.

Based on the Risk score of each patient, we successfully divided them into high- and low-risk subtypes using the median score. The clinical comparison between subtypes was summarized in Table 1. And there was no significant difference in age and gender features, suggesting the distribution of groups was harmonious. Regardless of in the training or test datasets, both the low-risk patients exhibited a longer median survival time than the high-risk cohorts in overall survival (OS) and progression free survival (PFS) analysis (Figures 3E,F). Moreover, compared to the high-risk cohorts, the low-risk patients manifested significant association with better clinical characteristics, including lower pathological grades, milder clinical stages and TNM stages (Figure 3G). The number of death patients was also increased with the increasing of Risk scores (Figure 4A) and the levels of above sites were consistent with the risk subtypes (Figure 4B).

**TABLE 1:** Comparison of clinical information between RNA modulation-related risk subtypes in STAD patients.

Variables	Low-risk (n = 145)	High-risk (n = 137)	P.value (Chi-square/Wilcoxon test)
Age (n/%)			0.850
≥65	82/56.55%	79/57.66%	
<65	63/43.45%	58/42.34%	
Gender (n/%)			0.910
Female	57/39.31%	53/38.69%	
Male	88/60.69%	84/61.31%	
Survival status (n/%)			<0.001***
Alive	116/80.00%	72/52.55%	
Dead	29/20.00%	65/47.45%	
Survival time/years	1.529 ± 1.207	1.139 ± 1.026	<0.001***
Grade (n/%)			<0.001***
G1-2	76/52.41%	30/21.90%	
G3	69/47.59%	107/78.10%	
Stage (n/%)			0.018*
I-II	87/60.00%	63/45.99%	
III-IV	58/40.00%	74/54.01%	
T stage (n/%)			<0.001***
T1-2	65/44.83%	37/27.01%	
T3-4	80/55.17%	100/72.99%	
N stage (n/%)			0.010**
N0	69/47.59%	45/32.85%	
N1-3	76/52.41%	92/67.15%	
M stage (n/%)			0.05
M0	136/93.79%	119/86.86%	
M1	9/6.21%	18/13.14%	

### Immunological features of ATIRE-related subgroups in stomach adenocarcinoma

To better explicate the potential mechanism of distinct ATIRE-related subtypes in GC patients, we further investigated their immunological characteristics comprehensively, including TME, ICI, and immune checkpoint expression. Notably, the results of ICI indicated that multiple immune cells were infiltrated in the tissues of low-risk GC patients, such as Macrophages, activated NK cells, CD4^+^ T-cells, CD8^+^ T-cells, Plasma cells and so on (Figures 4C,D). Conformably, TME analysis also demonstrated higher immune- and stromal-scores in low-risk patients with lower hub ATIRE-related sites than that of high-risk cohorts (Figures 4E,F). All these evidences suggested the immune-activated status in low-risk subgroups, which also interpreted its better prognostic outcomes. Interestingly, the low-risk patients possessed a lower expression of immune checkpoints than high-risk groups, indicating the immunotherapy might be potentially effective for high-risk patients (Figure 4G).

### Development of a prognostic nomogram system based on ATIRE-Risk score and clinical characteristics for stomach adenocarcinoma

**FIGURE 5:**
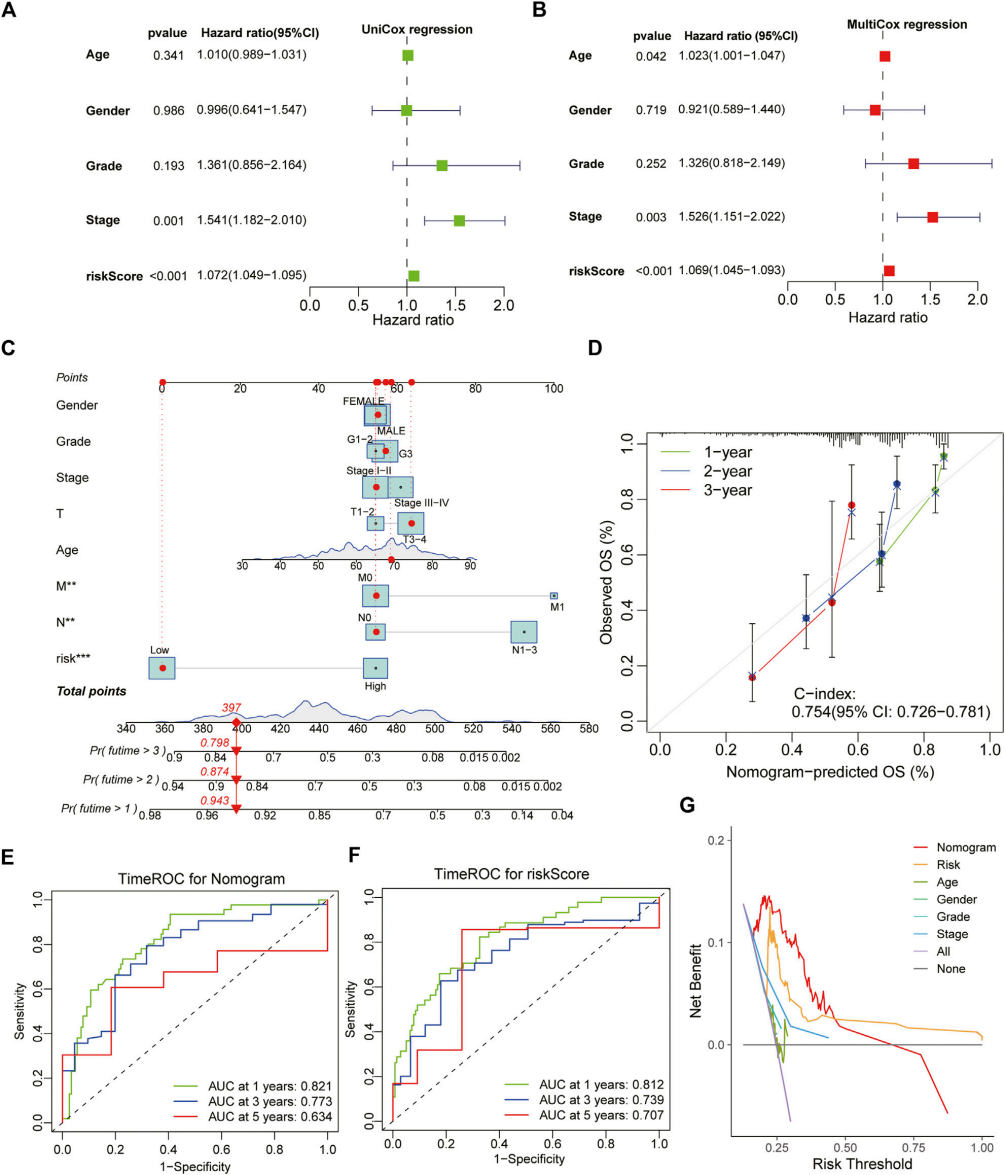
Construction and estimation of a combined prognostic model for STAD. **(A,B)** The forest chart showing the results of univariate Cox and multivariate Cox regression analysis with clinical characteristics and ATIRE-Risk score. **(C)** The combined nomogram for predicting the individual 1/3/5-year survival ratio for STAD patients. **(D)** The calibration curve of above established nomogram with C-index and 1/3/5-year survival ratio respectively. **(E,F)** Time-dependent ROC analysis of 1/3/5-years survival assessments for STAD patients using the nomogram model and ATIRE-Risk scores respectively. **(G)** The DCA analysis demonstrated the more significant role of Risk scores than other parameters in the nomogram model for GC patients.

Both univariate Cox and multivariate Cox regression analysis demonstrated that ATIRE-Risk score could serve as an independent risk-factor for the survival of GC patients with a high Hazard Ratio (HR) value, second only to pathological grades and clinical stages (Figures 5A,B). Combining the ATIRE-Risk scores and other essential clinical features, the nomogram was successfully constructed through multivariate Cox model to exactly predict the probability of 1/3/5-years survival for each GC patient (Figure 5C). This nomogram included the Risk scores, pathological grades, clinical stages, TNM stages, age and gender, and calibration curves further showed its good prediction capacity for GC patients (Figure 5D). The ROC analysis revealed the one/three/5-years AUC values of above nomogram were 0.821, 0.773, and 0.634, respectively while the AUC values of Risk scores also reached 0.812, 0.739, and 0.701 (Figures 5E,F). Interestingly, the DCA analysis also demonstrated the more significant role of Risk scores than other parameters in the nomogram model for GC patients (Figure 5G).

### Different transcriptomic pattern of ATIRE-related subtypes of stomach adenocarcinoma patients

**FIGURE 6:**
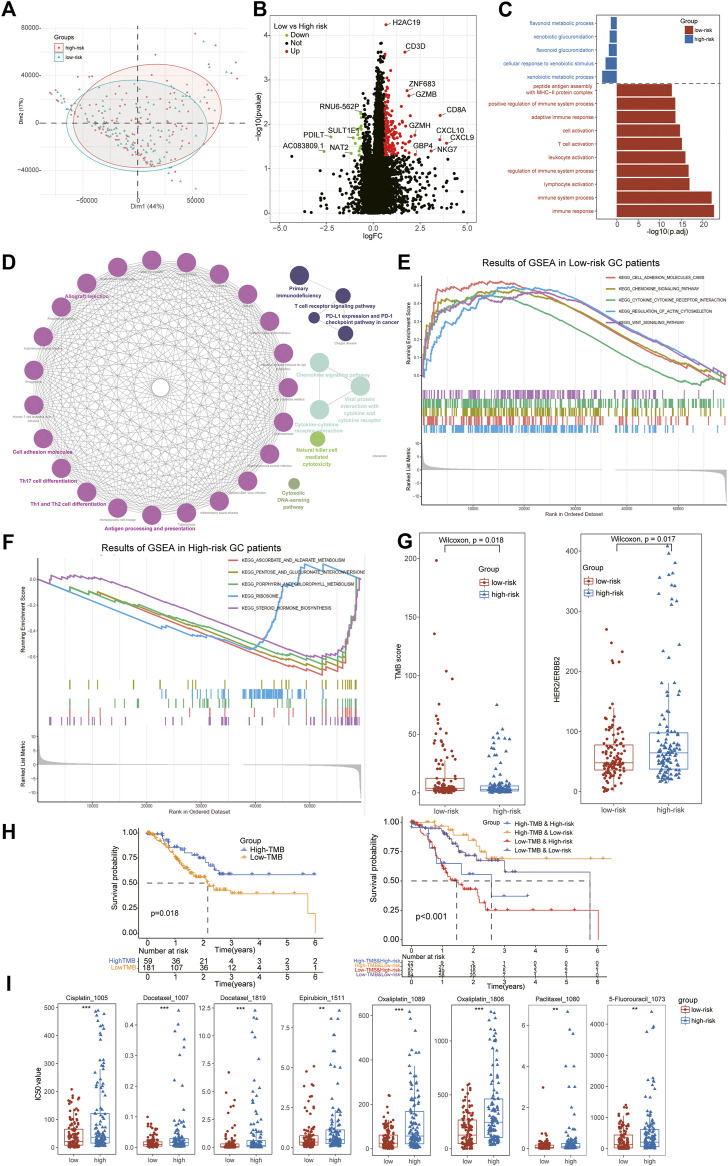
Identification of DEGs between subtypes and Evaluation of therapeutical susceptibility in STAD. **(A)** The PCA analysis showing significant differences in the RNA-seq transcription profiles between two ATIRE-Risk score related subgroups. **(B)** A total of 211 DEGs were identified including 189 DEGs for low-risk and 21 DEGs for high-risk patients. **(C)** Go enrichment analysis indicated that the low-risk DEGs were enriched in immune activation-related biological processes while the high-risk DEGs were enriched in metabolism-related biological processes. **(D)** The results of KEGG analysis found immune activation status of low-risk patients. **(E,F)** GSEA demonstrated the activation of above immune-related pathways in low-risk patients and metabolism-related pathways in high-risk patients. **(G)** The low-risk patients exhibited higher levels of TMB scores and lower expression of HER2, an index for targeted therapy in STAD patients. **(H)** The stratified survival analysis further indicated patients with low-risk and high-TMB scores exhibited the best prognosis outcomes. **(I)** All the anti-STAD chemotherapeutic drugs (including Cisplatin, 5-Fluorouracil, Docetaxel, Oxaliplatin, Epirubicin, and Paclitaxel) exhibited higher IC50 values in high-risk subgroups than that of low-risk STAD patients.

Furthermore, we also performed the differential expression analysis between low- and high-risk subtypes and a total of 211 DEGs were identified including 189 DEGs for low-risk and 21 DEGs for high-risk patients (Figure 6A,B). We noted several chemokines families and immune cells’ markers were the most significant genes for low-risk groups, such as CXCL9, CXCL10, CD3D, NKG7 and so on. The GO enrichment analysis indicated that the low-risk DEGs were enriched in immune activation-related biological processes including immune system process, lymphocyte activation and T cell activation, while the high-risk DEGs were enriched in metabolism-related biological processes including flavonoid metabolic process, xenobiotic glucuronidation and xenobiotic metabolic process (Figure 6C). The results of KEGG analysis further demonstrated immune activation status of low-risk patients, including Cell adhesion molecules, Natural killer cell mediated cytotoxicity, Chemokine signaling pathway, Cytokine-cytokine receptor interaction and Th1 and Th2 cell differentiation (Figure 6D). To clarify comprehensive functional characteristics of different subtypes, GSEA demonstrated the activation of above immune-related pathways in low-risk patients and metabolism-related pathways in high-risk patients (Figures 6E,F).

### Relationship of ATIRE-Risk score and tumor mutation burden and evaluation of chemotherapy susceptibility for stomach adenocarcinoma

Tumor mutation burden (TMB) has been reported as a significant element in alleviating the prognosis of multiple tumors through affecting the infiltration of CD8^+^ T-cells (Cristescu et al., 2018). To further investigate the potential relationship of TMB and ATIRE-Risk score, we compared the TME scores between high- and low-risk groups and it revealed a higher TMB scores in low-risk than that in high-risk patients (Figure 6G). The survival analysis displayed a longer median survival time in high-TMB than low-TMB subgroups, suggesting that TMB scores could be served as an independent prognostic factor for GC. In addition, the stratified survival analysis further indicated patients with low-risk and high-TMB scores exhibited the best prognosis outcomes, implying the role of TMB and Risk scores was synergistic for the prognosis of STAD (Figure 6H). These results suggested the potential negative correlation of TMB values and ATIRE-Risk scores, and their complementary effects for the forecast of prognosis in GC patients.

In present clinical guidelines, the expression of HER2 has been acknowledged as an indispensable index for targeted therapy, such as Trastuzumab, Parbolizumab and so on (Lordick et al., 2022). Hence, we also detected the expression of HER2 between different risk score subtypes and it revealed that high-risk patients exhibited a higher level of HER2, consistent with the expression of immune checkpoints (Figure 6G). Furthermore, anti-STAD chemotherapeutic drugs (including Cisplatin, 5-Fluorouracil, Docetaxel, Oxaliplatin, Epirubicin and Paclitaxel) also exhibited higher IC50 values in high-risk subgroups, implying these GC patients might obtain a poor curative response from the traditional chemotherapy (Figure 6I).

## Discussion

With rapid exacerbation and high mortality, the prognosis of STAD remains poor with postoperative recurrence and drug resistance due to individual abnormal genomic changes (Guo et al., 2019). Although the development of combination chemotherapy has been improved greatly, most STAD patients only obtain a mild survival amelioration due to chemotherapy resistance (Tan and Norhaizan, 2019). Novel targeted treatments provide an effective and individualized therapeutic option for resistant patients such as PD-1 inhibitors, tumor vaccines, and so on (Kim et al., 2018). However, even for the patients at the same clinical stages, the response to immunotherapy of individuals was still absolutely different in clinical practice. Therefore, the identification of a novel reliable index to evaluate the heterogenicity and therapeutical effects to immunotherapy for STAD is urgently required.

Recently, a majority of studies have constructed massive prognostic models for STAD patients via analyzing the transcriptome datasets, of which most studies focused on annotating the transcriptomics data into known characteristics of gene sets, such as Autophagy-related genes (Tian et al., 2022), Cuproptosis-related lncRNA (Feng et al., 2022), Pyroptosis-related signatures (Guan et al., 2022) and so on. However, there were still some limitations to expand their application in clinical practice due to the instability and collinearity of gene expression. Hence, screening a novel stabile biomarker for predicting the prognosis and therapeutic response in STAD patients. In this study, we first identified ATIRE-Risk scores based on the high associations between ATIRE sites with both OS and DFS in STAD, and further divided the STAD patients into two different subtypes with distinct clinical stages and immunological features including ICI, TME, and immune checkpoints expression. Interestingly, the low-risk patients exhibited milder clinical and pathological stages than the high-risk cohorts with a longer median survival time of OS and PFS. It revealed the low-risk patients were at mild disease status and exhibited a better prognosis in STAD.

To clear elaborate inner immunological difference, the results of immune analysis also demonstrated the activation condition of the low-risk patients with higher ESTIMAE scores and infiltration of massive immune-cells of inherent and adaptive immunity. These messages indicated that the activation of multiple immunity might be associated with mild status in low-risk STAD patients, consistent with the previous studies (Kwak et al., 2020). Although the prognosis of high-ATIRE score patients was relatively poor, higher expression levels of immune checkpoints were detected in high-risk patients than that of low-risk patients, suggesting these patients might be more sensitive to immunotherapy. In addition, we also identified some subtype-related DEGs and the functional enrichment also demonstrated the DEGs from low-risk cohorts were enriched in immune-related pathways, consistent with above results.

As a novel biomarker for immune-checkpoint treatments in multiple cancers, TMB has been reported to be used for predicting the survival outcomes of advanced GC patients after immunotherapy (Kim et al., 2020). In our study, we also detected significant increased TMB scores in low-risk subtypes and the survival analysis also demonstrated high-TMB patients exhibited a better survival prognosis for STAD. Our stratified results further revealed the congenerous effects between Risk and TMB scores, and the patients with low-risk and high-TMB scores displayed the optimal survival status, suggesting these two indexes were independent factors for the prognosis of STAD. For the drug susceptibility, the therapeutic responses to multiple combined chemotherapy were estimated from the GDSC database, including “CF”, “ECF”, “ELF”, “FAM”, and “DCF” chemotherapy regimen (Kim et al., 2022). Notably, anti-GC chemotherapeutic drugs (Cisplatin, 5-Fluorouracil, Docetaxel, Oxaliplatin, Epirubicin and Paclitaxel) all exhibited lower IC50 values in low-risk subgroups, suggesting these patients might obtain a better curative response from the combined chemotherapy of STAD. Interestingly, the high-risk patients exhibited higher levels of HER2, the objective of targeted therapy, consistent with the expressions of immune-checkpoints.

Finally, combined with other traditional clinical characteristics and ATIRE-Risk scores, we successfully established a useful nomogram system to exactly predict the one/three/5-year survival ratio of STAD with high AUC values (0.821, 0.773, and 0.634 respectively). In addition, calibration plots also confirmed its predictive effects for the survival of STAD patients with high Harrell’s C-indexes as 0.754 and DCA analysis showed that patients could obtain more net benefits to prognosis via applying the nomogram system when the risk threshold value was less than 0.5. Notably, whether for ROC estimation or DCA analysis, the ATIRE-Risk scores accounted for the most proportion of predictive efficiency, indicating that Risk score’s essential worthiness for the prognosis of STAD.

Nevertheless, there still remains some ineluctable limitations in the study. Firstly, the initial profile of ATIRE sires was obtained from the public databases and the analysis was still relatively insufficient. Different from the RNA-seq data, it is difficult to screen more external datasets to perform the validation and we could only divide the profile into training and test datasets to complete the validation. In addition, some results and fundings still need to be further investigated via other external studies and further validated by experiments *in vivo* or vitro. Finally, the application of our ATIRE-Risk scores was still lack of confirmation through clinical practices to be improved for predicting the prognosis of STAD.

## Conclusion

Overall, this study firstly identified a novel index called ATIRE-Risk score and divided the STAD patients into two different subtypes with clinical and immunological characteristics. Moreover, the Risk score exhibited significant predictive capability for the survival prognosis of STAD and potential insights for predicting the therapeutic responses via close relationships with TMB. Finally, combined with classical clinical features and ATIRE-Risk scores, we successfully established a promising nomogram system to accurately predict the 1/3/5-years survival ratio of STAD and this model was also estimated with high diagnostic efficiency and stable C-index with calibration curves. These significant ATIRE sites are promising to be further explored and might server as a novel therapeutic target for STAD treatment.

## Data Availability

The datasets presented in this study can be found in online repositories. The names of the repository/repositories and accession number(s) can be found in the article/Supplementary Material.
